# Similarity-Based Segmentation of Multi-Dimensional Signals

**DOI:** 10.1038/s41598-017-12401-8

**Published:** 2017-09-27

**Authors:** Rainer Machné, Douglas B. Murray, Peter F. Stadler

**Affiliations:** 1 Institute for Synthetic Microbiology, Cluster of Excellence on Plant Sciences (CEPLAS), Heinrich Heine University Düsseldorf, Universitätsstraße 1, D-40225 Düsseldorf, Germany; 2 Institute for Advanced Biosciences, Keio University, Tsuruoka, Yamagata 997-0017 Japan; 3 Bioinformatics Group, Department of Computer Science, Interdisciplinary Center for Bioinformatics, German Centre for Integrative Biodiversity Research (iDiv) Halle-Jena-Leipzig, Competence Center for Scalable Data Services and Solutions, and Leipzig Research Center for Civilization Diseases, University Leipzig, Härtelstrasse 16-18, D-04107 Leipzig, Germany; 4 Max Planck Institute for Mathematics in the Sciences, Inselstraße 22, D-04103 Leipzig, Germany; 5 Fraunhofer Institute for Cell Therapy and Immunology, Perlickstrasse 1, D-04103 Leipzig, Germany; 6 Department of Theoretical Chemistry of the University of Vienna, Währingerstrasse 17, Vienna, A-1090 Austria; 7 Center for RNA in Technology and Health, Univ. Copenhagen, Grønneg ardsvej 3, Frederiksberg C, Denmark; 8 Santa Fe Institute, 1399 Hyde Park Road, Santa Fe, NM 87501 USA

## Abstract

The segmentation of time series and genomic data is a common problem in computational biology. With increasingly complex measurement procedures individual data points are often not just numbers or simple vectors in which all components are of the same kind. Analysis methods that capitalize on slopes in a single real-valued data track or that make explicit use of the vectorial nature of the data are not applicable in such scenaria. We develop here a framework for segmentation in arbitrary data domains that only requires a minimal notion of similarity. Using unsupervised clustering of (a sample of) the input yields an approximate segmentation algorithm that is efficient enough for genome-wide applications. As a showcase application we segment a time-series of transcriptome sequencing data from budding yeast, in high temporal resolution over ca. 2.5 cycles of the short-period respiratory oscillation. The algorithm is used with a similarity measure focussing on periodic expression profiles across the metabolic cycle rather than coverage per time point.

## Introduction

The segmentation problem consists in finding a piece-wise constant approximation to a function defined on a 1-dimensional independent variable, which may be e.g. time or a genomic coordinate. The problem arose early in the -omic era in the context of detecting copy number variations in array comparative genomic hybridization (CGH) data and for determining transcripts from RNA expression data measured by tiling arrays. For a 1-dimensional readout, coverage, it can be solved by dynamic programming^1,2^. With the rapidly increasing flood of high throughput data the problem was generalized to segmenting vector-valued data such as multiple transcriptomes or CGH data from an entire cohort of patients. Again a dynamic programming solution can be found^3^.

In many applications one is not only interested in a segmentation but at the same time attempts to infer clusters of measurements that behave in similar way. This problem has typically been tackled by combining an expectation-maximization (EM) step to train a model that is then used to produce the segmentation. A hybrid of EM and the dynamic programing algorithm was used for multidimensional CGH data^3^, in a transcriptomics context hidden semi-Markov models were used to deal with the directional nature of strand-specific transcriptome data^4^.

A particularly important version of the problem is chromatin segmentation, i.e., the assignment of biologically interpretable functional annotations based on a pattern of histone modifications typically measured by ChIP-seq. ChromaSig^5^ then uses a greedy algorithm to extend seed patterns to determine segments. ChromHMM^6,7^ and EpiCSeg^8^ are based on a hidden Markov models (HMM), Segway^9^ uses Dynamic Bayesian Network, and bidirectional HMMs are proposed to model the assignment of binarized (presence/absence) data or ChIP-Seq signal intensities or read counts to chromatin states^10^. The models are trained on a subset of the data and then a Viterbi algorithm or its equivalent is then used to decode the complete data track, i.e. to produce a segmentation of the complete data set.

A closely related segmentation problem is the detection of differentially methylated regions (DMRs) from whole genome bisulfite sequencing data. While some tools use simple heuristics to aggregate differentially methylated CpGs into DMRs (e.g. BS-seq^11^, MOABS^12^, or MethylSig^13^). More recently, HMM based methods such as HMM-Fisher^14^ have been introduced.

Another important variation on the theme are reference-based transcriptome assemblers such as cufflinks^15^, TruHMM^16^, ORA^17^, or TransComb^18^. These tools, however, do not solve a general segmentation problem. The HMM of TruHMM, for instance only separates expressed from non-expressed states. Similarly, the determination of intron-exon boundaries can be viewed as a particular, specialized segmentation problem. We refer to reference^19^ for a recent review and comparison.

HMMs and similar stochastic models are attractive theoretically because (i) such models inherently define segmentations, (ii) they provide a consistent statistical framework, and (iii) they are capable of computing measures of confidence for the results. The EM training, however, can be computationally rather expensive for large data sets. Furthermore, it requires a user-defined number of labels (such as chromatin states) and it depends on the vector structure of the data (such as position-wise counts or presence/absence information for each position and each transcriptome or ChIP-seq experiment. It is of interest therefore, to investigate whether the expensive model training can be replaced by computationally more efficient heuristics. This avenue has been explored e.g. in metilene^20^, which uses a fast heuristic to propose differentially methylated regions that are tested for significance only in a second step.

In this contribution we describe a general purpose segmentation method for essentially arbitrary linearly ordered data. It relies only on a similarity function and proceeds in three separate steps: (i) The measurements are clustered using an unsupervised clustering method so that each measurement is replaced by membership to one of the clusters and possibly an additional “garbage bin”; (ii) Cluster-cluster and/or position-cluster similarity measures are calculated; (iii) The resulting coarse-grained series of cluster IDs is segmented using an exact dynamic programming algorithm.

The data set that motivated the work presented here is a time-series of 24 RNA-seq transcriptomes covering about two and a half cycles of oscillatory growth dynamics (‘metabolic cycle’) in budding yeast (*Saccharomyces cerevisiae*) continuous culture. In this experimental system, a large majority of protein-coding transcripts showed dynamic (oscillatory) abundance signals in earlier hybridization-based transcriptome measurement (‘microarrays’) that are reproducible across a variety of strains and conditions^21–23^.

The budding yeast genome is compact, i.e densely packed with transcripts with a low frequency of spliceosomal introns^24^, and expresses a large number of antisense and long non-coding RNAs^25–27^ that often partially overlap other transcriptional units. This makes inference of yeast transcripts difficult for the standard tools, which are optimized for mammalian transcriptomes and use splice junctions as an import source of information. Moreover, during the metabolic oscillation the transcriptome is highly dynamic, so that different time points cannot be simply pooled before transcripts are characterized. Taken together, segmentation thus would seem to be a more prudent approach.

The correlation between consecutive time points and the usually well-defined phase of the expression changes w.r.t. to the metabolic cycle on the one hand, and the large variations of coverage between different loci of the same annotated transcript on the other hand, strongly suggest not to focus on the expression levels in individual experiments but on the position-wise *pattern* of the time-course for the purpose of segmentation. A clustering strategy for such oscillatory transcriptome data based on selected and scaled components of the discrete Fourier Transform (DFT) of the time-series was introduced for corresponding microarray data^23^, where it was used to identify groups of genes with consistent temporal profiles. This strategy has proved superior in a scan of various data transformation and clustering strategies for similar data, diurnal expression profiles in a cyanobacterium^28^. Here we employ the same technique to quantify similarities of temporal expression changes at single nucleotide resolution. This amounts to abandoning the vector space structure of the data and thus renders existing segmentation tools inapplicable. We therefore consider the segmentation problem in a more general formal setting, which assumes that the position-wise signal “lives” in a similarity space, a mathematical construct even more general than the more familiar metric spaces. In particular, we do not any longer assume that there is a concept of mean values or averages.

In this contribution we first summarize the necessary theoretical background to formulate the segmentation problem in arbitrary similarity spaces. We then show that it can be solved by means of dynamic programming in a manner similar to more restricted segmentation problems. Finally, we focus on the application to yeast transcriptome time series to demonstrate the practical applicability of our approach.

## Theory

We start from a signal $x\mathrm{:[1,}\,\mathrm{...,}\,n]\to {\mathbb{X}},\,i\mapsto {x}_{i}$ that is measured on a finite sequence of consecutive points, which without loss of generality we can label $i\,=\,\mathrm{1,}\ldots ,n$. For the domain ${\mathbb{X}}$ of measured data we assume only that the similarity between two measurements can be quantified. That is, there exists a similarity function $\sigma :{\mathbb{X}}\times {\mathbb{X}}\to {\mathbb{R}}$ such that for all $u,v\in {\mathbb{X}}$ holds

(s0)    $$\sigma (u,u)\ge 0$$;

(s1)    $$\sigma (u,v)=\sigma (v,u)$$;

(s2)    $$\sigma (u,u)\ge \sigma (u,v)$$;

The pair $({\mathbb{X}},\sigma )$ satisfying (s1) and (s2) is a *similarity space*. For completeness of the presentation we note that two additional axioms,

(s3)    $$\sigma (u,v)+\sigma (v,w)\le \sigma (u,w)+\sigma (v,v)$$;

(s4)    $$\sigma (u,v)=\sigma (u,u)=\sigma (v,v)$$ iff $$u=v$$.

are satisfied for all $u,v,w\in {\mathbb{X}}$. Axiom (s3) corresponds to the triangle inequality. If axiom (s4) holds in addition, there is an equivalent metric space with distance function ${d}_{\sigma }(u,v)=\sigma (u,u)-\sigma (u,v)$. We will require neither condition here since similarity functions satisfying (s1) and (s2) already introduce sufficient structure on ${\mathbb{X}}$ for well-defined clustering and classification algorithms, see e.g.^29–33^. The $k$-medoids strategy^34,35^, for example, is a generalization of the well-known $k$-means clustering that only requires similarity data, see also^36,37^ for more recent developments.

A *segmentation* ${\mathfrak{S}}:=({i}_{1},\ldots ,{i}_{\ell -1})$ with $1\,=\,{i}_{0} < {i}_{1} < \ldots  < {i}_{\ell }=n$ is a sequence of $\ell -1$ *jump points* or *break points* that partitions $\mathrm{[1,}\,n]$ into $\ell$ non-empty, consecutive intervals ${I}_{h}:=[{i}_{h-1},{i}_{h}]$ for $1\le h\le \ell$. The coarsest segmentation, for $\ell =1$, consists only of the interval $\mathrm{[1,}\,n]$ itself, while the finest segmentation assigns each point to a distinct interval, ${I}_{h}=\{h\}$. A given segmentation ${\mathfrak{S}}$ defines sets ${{\mathscr{J}}}_{h}:=\{{x}_{i}\in {\mathbb{X}}|i\in {I}_{h}\}$ of signals observed in the corresponding interval.

Good segmentations are distinguished from bad ones by a measure of the similarities of signal values within each set ${{\mathscr{J}}}_{h}$. Since ${{\mathscr{J}}}_{h}$ is uniquely determined by the interval ${I}_{h}$ we can think of the scores as a functions $f({I}_{h})$ of the intervals. The quality of a particular segmentation ${\mathfrak{S}}$ is then $f({\mathfrak{S}})={\sum }_{{I}_{h}\in {\mathfrak{S}}}\,f({I}_{h})$. We now can define the segmentation problem in one of the following ways: (i) We fix a number $\ell$ of desired segments, or (ii) we fix a cost $M$ for each jump. In first case we ask for the segmentation with $\ell$ non-empty intervals that maximizes $f({\mathfrak{S}})$. In the second case we allow $\ell$ to vary and penalize the use for more distinct intervals then necessary by maximizing ${\sum }_{h}\,f({I}_{h})-M(\ell -\mathrm{1)}$.

It is easy to see that both problems can be solved by essentially the same dynamic programming approach. Denote by ${S}_{i,h}$ the optimal segmentation of the sub-problem on the points $\mathrm{[1,}\,\mathrm{...,}\,i]$ that consists of exactly $h$ non-empty intervals. For brevity we write ${f}_{i,j}:=f([\mathrm{i,}\,\mathrm{...,}\,j])$. These values satisfy the recursion

1 $$\begin{array}{lll}{S}_{k\mathrm{,1}} & = & {f}_{\mathrm{1,}k}\\ {S}_{k,h} & = & \mathop{\max }\limits_{h-1\le j < k}{S}_{j,h-1}+{f}_{j,k}\end{array}$$

In the first case, the optimal solution is found as ${S}_{n,\ell }$, for the other variant, one needs to determine ${\max }_{h}\,{S}_{n,h}-(h-\mathrm{1)}M$. An optimal segmentation is obtained by backtracing from these entries in either case. Equ. ((1)) provides a polynomial time solution in ${O}({n}^{2}\ell )$ or $O({n}^{3})$ time and $O(n\ell )$ or $O({n}^{2})$ space, assuming that the scores ${f}_{j,k}$ can be computed fast enough.

The structure of the cost function $f$ is not arbitrary. Most importantly breaking up an interval into two parts must not make the fit worse, i.e., ${f}_{i,k}\le {f}_{i,u-1}+{f}_{u,k}$ must hold for all $i < u\le k$. This condition implies ${f}_{i,k}\le {\sum }_{u=i}^{k}\,{f}_{u,u}$ and strongly suggests to consider scores of form

2 $$f({I}_{h}):=\sum _{i\in {I}_{h}}\sigma ({x}_{i},{{\mathscr{J}}}_{h})$$

where $\sigma (x,{\mathscr{J}}\,)$ measures the similarity of $x\in {\mathbb{X}}$ with a set ${\mathscr{J}}\subseteq {\mathbb{X}}$. In general similarity spaces there are at least two natural choices for $\sigma (x,{\mathscr{J}}\,)$. One may represent the set ${\mathscr{J}}$ by a “representative point” ${x}_{{\mathscr{J}}}\in {\mathscr{J}}$ and define $\sigma (x,{\mathscr{J}}\,):={\sigma }_{m}(x,{x}_{{\mathscr{J}}})$. The most natural choice of a representative object is the medoid, which has the property that the total similarity ${\sum }_{y\in {\mathscr{J}}}\sigma (y,{x}_{{\mathscr{J}}})$ is maximal. Alternatively, the average similarity ${\sigma }_{a}(x,{\mathscr{J}}\,):\,=\,\mathrm{(1/|}{\mathscr{J}}|){\sum }_{y\in {\mathscr{J}}}\sigma (x,y)$ can be used. The nave evaluation of these measures is quadratic in the length of the interval, and hence would slow down the evaluation by another order of magnitude. More efficient evaluations are easily available, however (Appendix A in Supplementary Data).

Even with an efficient evaluation of ${f}_{i,j}$ the performance bounds on equ ((1)) are expensive for genome-wide applications. The basic idea to devise a much faster approximate solution is to replace the sets ${{\mathscr{J}}}_{h}$ of measurements within an interval with a precomputed clustering of the measurement comprising $N$ clusters ${{\mathscr{C}}}^{\alpha }\subseteq {\mathbb{X}}$, $\alpha \,=\,\mathrm{1,}\ldots ,N$. There is no restriction on the definition of these clusters. In particular the clusters may overlap or leave parts of the data set uncovered. The only formal requirement is that the similarity $\sigma$ between a point $x\in {\mathbb{X}}$ and a cluster ${{\mathscr{C}}}^{\alpha }$, introduced above, can be computed.

Denote by ${S}_{i,\alpha }$ the optimal score for segmentation of the interval [1, $\mathrm{...,}\,i]$ that assigns the last position $i$ to cluster ${{\mathscr{C}}}^{\alpha }$, and let $s(i,k,\alpha )$ denote a scoring function that measures the total similarity of all positions in the interval $[\mathrm{i,}\,\mathrm{...,}\,k]$ to the cluster $\alpha$. Again, we write $M$ for the penalty incurred by introducing and additional interval. Then ${S}_{k,\alpha }$ satisfies the recursion

3 $${S}_{k,\alpha }\,=\,\mathop{\max }\limits_{i\le k}\,\mathop{\max }\limits_{\beta \ne \alpha }{S}_{i-\mathrm{1,}\beta }+s(i,k,\alpha )-M$$

with the initialization (basis case) $S\mathrm{(0,}\,\beta )\,=\,M$ for all $\beta$, ensuring that ${S}_{i,\alpha }\ge s\mathrm{(1,}\,i,\alpha )$. This can be evaluated in $O({n}^{2}{N}^{2})$ time and $(nN)$ space. The optimal segmentation is then obtained by standard backtracing from the entry realizing the optimal score ${\max }_{\alpha }\,{S}_{n,\alpha }$.

Several scoring functions $s(i,k,\alpha )$ can be conceived. On the one hand, one might want to match the original segmentation problem as closely as possible. This amounts to using a score of the form

4 $$s(i,k,\alpha )=\sum _{j=i}^{k}\tilde{\sigma }({x}_{j},{{\mathscr{C}}}^{\alpha })$$

where $\tilde{\sigma }$ is either the average similarity between ${x}_{j}$ and the cluster ${{\mathscr{C}}}^{\alpha }$ (corresponding to ${\sigma }_{a}$ in the original problem) or the similarity between ${x}_{j}$ and the medoid point of ${{\mathscr{C}}}^{\alpha }$ corresponding to the ${\sigma }_{m}$ measure. The latter is particularly appealing if e.g. a $k$-medoid method was used for clustering. If a divisive clustering method was used, then usually no natural representative is available, and the average similarity score may be the natural choice. Of course, if $\sigma$ is derived from a vector space, i.e., centroids ${\bar{x}}_{\alpha }:\,=\,\mathrm{(1/|}{{\mathscr{C}}}^{\alpha }|){\sum }_{x\in {{\mathscr{C}}}^{\alpha }}x$ are well defined, these are the natural replacement for medoids.

Alternatively, we can determine the cluster ${{\mathscr{C}}}_{i}$ that fits best to ${x}_{i}$, i.e., ${{\mathscr{C}}}_{i}={\rm{\arg }}\,{{\rm{\max }}}_{\alpha }\sigma ({x}_{i},{{\mathscr{C}}}^{\alpha })$. It is not necessary to assign all measurements to a cluster. Instead, we may place a measurements ${x}_{i}$ into a “garbage bin” or “nuisance cluster” ${{\mathscr{C}}}^{0}$ if $\sigma ({x}_{i},{{\mathscr{C}}}^{\alpha }) < \theta$ for all regular clusters, i.e., $1\le \alpha \le N$ and some threshold similarity value $\theta$. In this setting, the scoring function is naturally constructed as

5 $$s(i,k,{\mathscr{C}})=\sum _{j=i}^{k}Q({{\mathscr{C}}}_{j},{\mathscr{C}})$$

where $Q({\mathscr{C}},{\mathscr{D}})$ is an, in principle arbitrary, similarity measure for the two clusters. The most basic choice is $Q({\mathscr{C}},{\mathscr{C}})=1$ and $Q({\mathscr{C}},{\mathscr{D}})=a < 0$ for ${\mathscr{C}}\ne {\mathscr{D}}$.

For the specific application scenario of transcriptome time series we found similarity measures derived from the Pearson correlation of a discrete Fourier transformation of the expression time series particularly useful, and equations (4) and (5) are implemented as scoring functions icor and ccor as outlined below (section ‘Implementation & Application’).

There are several possibilities to treat scattered outliers or special cases such a non-expressed locations. One approach is to use a smaller penalty value ${M}_{0}$ instead of $M$ for jumps to the “nuisance cluster” to allow such values to accumulate in short’special’ segments. Another plausible parametrization for a “nuisance cluster” is to simply set $s(i,k,\,\mathrm{0)}=M$, compensating the penalty $M$ for the jump to ${{\mathscr{C}}}^{0}$ thus adding no contribution to the similarity score at all for the nuisance cluster.

To make the parameters easier to interpret, it can be convenient to use a scaled version so that $\tilde{\sigma }$ takes values between $0$ and $1$. The jump penalty parameter $M$ then serves as a lower bound on the length of a segment.

We observe, finally, that with any of the choice of the scoring functions discussed above, it not necessary to store or precompute all values of $s(i,k,{\mathscr{C}})$. For every real cluster and $i\mathrm{ > 1}$ we have

6 $$s(i,k,\alpha )=s\mathrm{(1,}\,k,\alpha )-s\mathrm{(1,}\,i-\mathrm{1,}\,\alpha )$$

Hence it suffices to compute the $O(nN)$ entries of $s\mathrm{(1,}\,k,\alpha )$ for all $k$ and $\alpha$.

The quadratic running time suggests to employ a heuristic pre-segmentation step that reduces the problem to smaller pieces at positions where break points are obvious such as longer genomic regions without expression or histone modification signals. Alternatively, the running time can be reduced by cutting the input into overlapping pieces with a size a few times larger than some upper bound on the expected segment size.

## Implementation and Application

The algorithm was implemented in R, where the work-horse scoring routines are written in C++ *via* Rcpp^38^, and is available as an R library from github (https://github.com/raim/segmenTier). The state of the code used herein is tagged as release v0.1. The library additionally implements the time-series preprocessing (data transformations, including the DFT) and clustering strategies used in this presentation. The implementation is specifically equipped for batch calculations over different input clusterings and parameter settings, to scan for optimal parameters for the given segmentation objective.

The package is geared towards use from command-line scripts in parallel computing environment, and these command-line scripts are available from the *segmenTools* code repository and R library (https://github.com/raim/segmenTools). This library provides all functionality used for the comparative evaluation of segmentation characteristics and quality.

### RNA-seq Time-Series Clustering and Similarity Measures

The time-series of read-counts for each nucleotide (see ‘Additional Methods’) were processed as previously described for micro-array based periodic expression data^23^ with data- and problem-specific adaptations. The following processing pipeline is implemented in segmenTier as function processTimeseries. A Discrete Fourier Transform was applied to the raw (trafo = raw) or arcsinh-transformed (trafo = ash) time-series, and all components were amplitude-scaled (use.snr = TRUE) as described^23^. High-frequency components were discarded and only the informative components 1–7 (dft.range = 1:7) were selected for clustering. Components 2–7 carry the main information of the time-series profiles. Unlike previously, we included the first DFT component, which provides information on the temporal mean of expression. This allowed for a better separation of very close adjacent segments with otherwise similar temporal profiles. To avoid segmentation by the mean expression alone, this component was separately scaled by the arcsinh transformation (dc.trafo = ash). All non-expressed positions were assigned to the nuisance cluster.

The transformed data was then clustered by the base R implementation of $k$-means using the Hartigan-Wong algorithm^39^, *via* the package’s clusterTimeseries function. This function also calculates the similarity matrices for the scoring functions, specifically Pearson correlation coefficients between cluster means for ccor or between each position and cluster means for icor. For the latter we implemented the algorithm in C++ (*via* Rcpp) for efficiency. After clustering another filtering step proved useful for scoring function ccor. Positions with a correlation coefficient below a threshold $\theta$ (nui.thresh = 0.6) to their cluster center were re-assigned to the nuisance cluster. See Appendix A for the definition of $\theta$ in context.

### Scoring Functions

The simple scoring $Q({\mathscr{C}},{\mathscr{D}})=1$ or $a$ depending on whether ${\mathscr{C}}$ and ${\mathscr{D}}$ are equal or distance is available as ccls scoring in segmenTier with default value $a=-1$ (equ. (5)).

For vector-like data such as the DFT of the time series of yeast transcriptomes that motivated this work, the Pearson correlation coefficient $corr({\bar{x}}_{{\mathscr{C}}},{\bar{x}}_{{\mathscr{D}}})$ is a natural choice of the similarity measure. As it turned out, a transformation that reduced the influence of moderate positive or negative correlations improved the segmentation performance. To this end we used transformation ${\phi }_{\varepsilon }:[-\mathrm{1,}+\mathrm{1]}\to [-\mathrm{1,}+\mathrm{1]:}t\mapsto sign(t){t}^{\varepsilon }$. Note that ${\phi }_{1}$ is the identity. For large $\varepsilon$ the transformation approaches ${\phi }_{\infty }(-\mathrm{1)}=-1$, ${\phi }_{\infty }\mathrm{(1)}=1$, and ${\phi }_{\infty }(t)=0$ for $-\mathrm{1 < }t\mathrm{ < 1}$.

segmenTier implements both $\tilde{\sigma }({x}_{i},{{\mathscr{C}}}^{\alpha })={\phi }_{\varepsilon }(corr({x}_{i},{\bar{x}}_{{{\mathscr{C}}}^{\alpha }}))$ and $Q({\mathscr{C}},{\mathscr{D}})={\phi }_{\varepsilon }(corr({\mathscr{C}},{\mathscr{D}}))$ as options icor and ccor. The transformation parameter $\varepsilon$ is available as parameter E.

The ‘nuisance cluster’ correlation $\nu$ is available as parameter nui in segmenTier. For our test data $\nu  > 1$ proved useful to enforce breaks between close adjacent transcripts and cut ends at the transition to noisy low-expressed regions. Finally, the length-penalty $M$ and the nuisance-specific ${M}_{0}$ are available as parameters M and Mn in segmenTier.

Two package demos demonstrate the usage of the package and effects of parameters. The first (segment_test) uses an artificial data set to demonstrate the low level interface to the algorithm, function segmentClusters, and how to construct similarity matrices for scoring functions icor and ccor. It further shows that variations in scoring matrix calculation and back-tracing affect only the discrete scoring function ccls (Supp. Fig. S1). The second demo (segment_data) reproduces Fig. 3 and S4 of this article and demonstrates the usage of higher-level interfaces and S3 data structures for processing of time-series, including simple wrappers for time-series transformation, clustering, segmentation and plotting.

## Additional Methods

### Data Acquisition

The yeast strain used in this study was IFO 0233 and continuous culture experiments were carried out as previously described^40^. Total RNA was extracted^41^ from 24 samples taken every 4 min, covering ca. 2.5 cycles of the respiratory oscillation (Supp. Fig. S2), and DNA removed (RNase-Free DNase Set, Qiagen, Japan). Total RNA had an RNA integrity number > 7 and 260 nm:230 nm and 260 nm:230 nm ratios > 2.14. Strand specific cDNA libraries were created using the dUTP method^42,43^ and sequencing was carried out on an Illumina 1 G sequencer. All cDNA libraries were generated and sequenced by BGI, China.

RNA-seq reads were mapped against yeast S288C genome (release R64-1-1) using segemehl (version 0.1.4)^44^ with default parameters and spliced read mapping enabled. Initially unmatched reads were mapped again using the remapping tool from the segemehl package and the resulting files were merged. Coverage (read-counts per nucleotide) was normalized for total library size and was stored in a bedgraph file for further analysis.

### Pre-Segmentation

The algorithm performance was improved by using areas largely devoid of RNA-seq coverage as “safe anchors” for pre-segmentation. To this end we calculated for each genomic position $i$ the number ${n}_{i}$ of time-points with any signal at all, i.e., ${x}_{i}\ne \overrightarrow{o}$. A moving average ${\tilde{n}}_{i}/d$ of this signal, scaled by the total number of measured time points $d$ (here 24), is then used to determine non-expressed region. For the yeast data we used a window-size of avg = 1000 nt for the running average, and required an interval of length $1000$ nt where the moving average does not exceed the threshold ${\tilde{n}}_{i}/d\le \mathrm{1/3}$ (achieved by option minrd = 8, indicating 8 of 24 time points) for the average number of nucleotides with any expression signal at all. To avoid the removal of weakly expressed regions close to non-expressed intervals, we extended pre-segments on both ends until the ${\tilde{n}}_{i}/d$ signal, computed with a much narrower moving average over only favg = 100 nt, dropped to $0$. Finally, adjacent pre-segments with a length of less than minsg = 1000 nt were fused with their neighbors, and segments spanning chromosome ends were split there. The pre-segmentation heuristics is implemented in the R script wrapper presegment.R and distributed with the segmenTools package.

**Figure 1:**
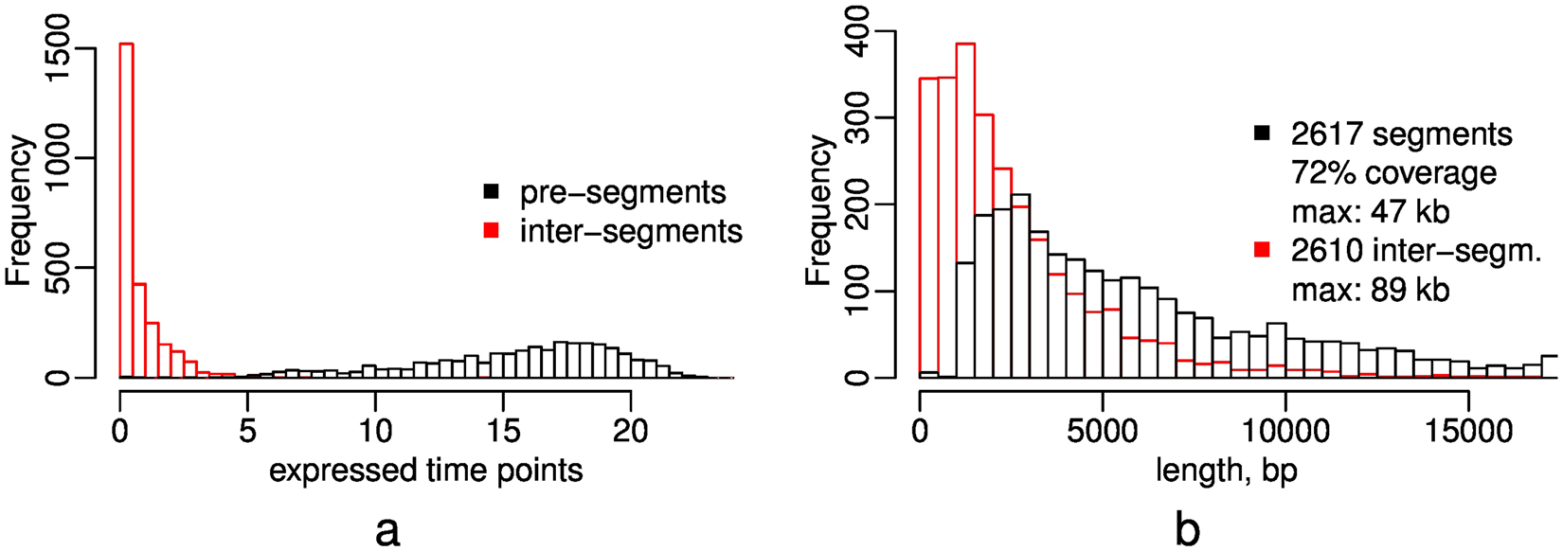
Length distributions of the pre-segmentation. Distribution of mean number of expressed time points, read-count presence $\bar{n}=(i-j+{\mathrm{1)}}^{-1}{\sum }_{j}^{i}{n}_{k}$, (**a**) and lengths (**b**) of genomic intervals of the final pre-segments (black) and inter-segment regions (red).

Application of the pre-segmentation heuristic to the yeast data set produced $2617$ pre-segments between 1 and 47 kb, and in total covering 72% of the genome (Fig. 1). The majority of $2610$ inter-segment regions is small; the largest (89 kb) corresponds to the mitochondrial genome and was skipped due to the specific complex structure (large introns, overlapping ORF) of the mitochondrial transcriptome. Both the expressed pre-segments and the inter-segments, potentially containing lowly expressed smaller transcripts, were then used as input for segmentation.

### Parameter Scan and Optimization

**Figure 2:**
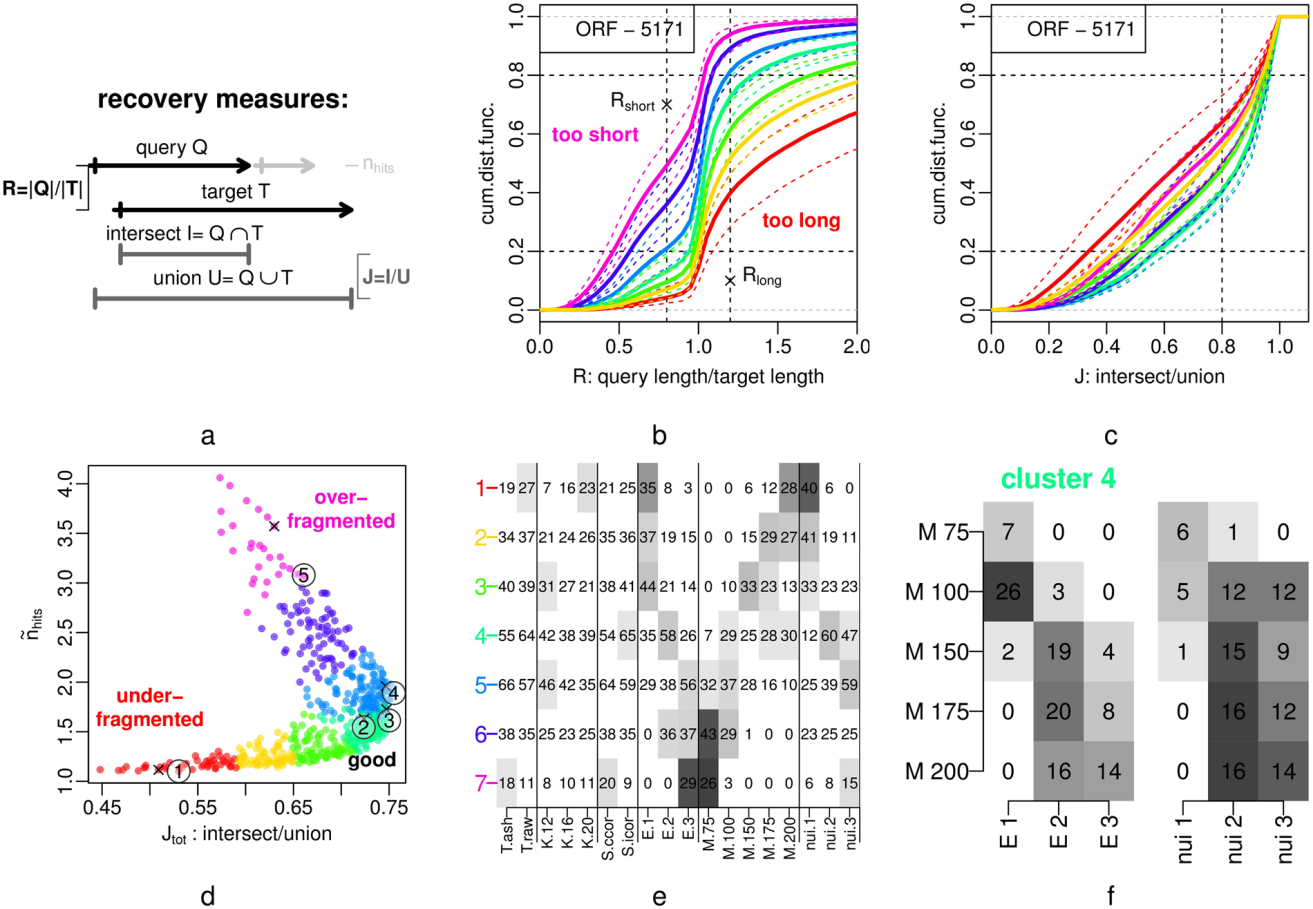
Parameter Scan & Selection: ORF Transcript Recovery. 540 segmentations were calculated with varied parameters, and segments (queries $Q$) from each segmentation were tested against a set of 5171 annotated ORF transcripts (targets $T$). (**a**) Graphical definition of recovery measures: ratio $R$, Jaccard index $J$ and the number of segments per target ${n}_{hits}$ (also see Methods section). Only the best matching hits for each target were used for calculation of $R$ and $J$. (**b**) Empirical cumulative distribution function (ECDF) of query-target length ratios $R$: mean values of the clusters (solid lines) defined in panel d, and the full spread of values in each cluster (dashed lines). A segmentation where many query segments that are longer than their matching ORF transcript target ($R\,\mathrm{ > \; 1}$) is interpreted as under-fragmentation of the data (e.g. the ‘too long’ red segmentations), while segments which are shorter than their target ($R < \mathrm{1)}$ point to over-fragmentation (e.g. the ‘too short’ magenta segmentations). (**c**) ECDF of the best hit Jaccard indices, plotted as in panel b. (**d**) The total Jaccard index ${J}_{tot}$ of best-matching pairs (x-axis) and ${\tilde{n}}_{hits}$, the average number of segments per target (y-axis). Numbers indicate the example segmentations in Fig. 3, the slightly shifted ‘x’ indicate the same parameters but with scoring function ccor instead of icor. The coloring of segmentations is derived from a PAM clustering of the ratios ${R}_{short}$ and ${R}_{long}$ (vertical lines in panel b), and ${J}_{tot}$ and ${\tilde{n}}_{hits}$. (**e**) Frequency distribution of parameters (T: time-series processing, K: number of clusters, S: scoring function, E: similarity transformation exponent $\varepsilon$, M: length-penalty parameter $M$, nui: nuisance cluster correlation $\nu$) in the PAM clusters; the gray-scale is derived from the p-values ($-log\mathrm{2(}p)$) of Fisher’s exact tests of the overlaps and indicates enrichment. This provides an overview of parameter effects on optimization criteria. Detailed effects for each parameters are shown in Supp. Fig. S3a. (**f**) Frequency distributions of parameter combinations in the ‘optimal’ cluster 4, gray-scale is derived from the shown frequencies; see Supp. Fig. S3b for other clusters.

In order to test the quality of the segmentation, we used a set of 5171 experimentally defined transcripts that were assigned to open reading frames (ORF-T)^26^, as available from the yeast genome database^45^ (file Xu_2009_ORF-Ts_V64.bed). With our segmentation as the query $Q$ and annotated ORF-T as the target $T$, we calculated three parameters for each target segment, graphically defined in Fig. 2a.

**Figure 3:**
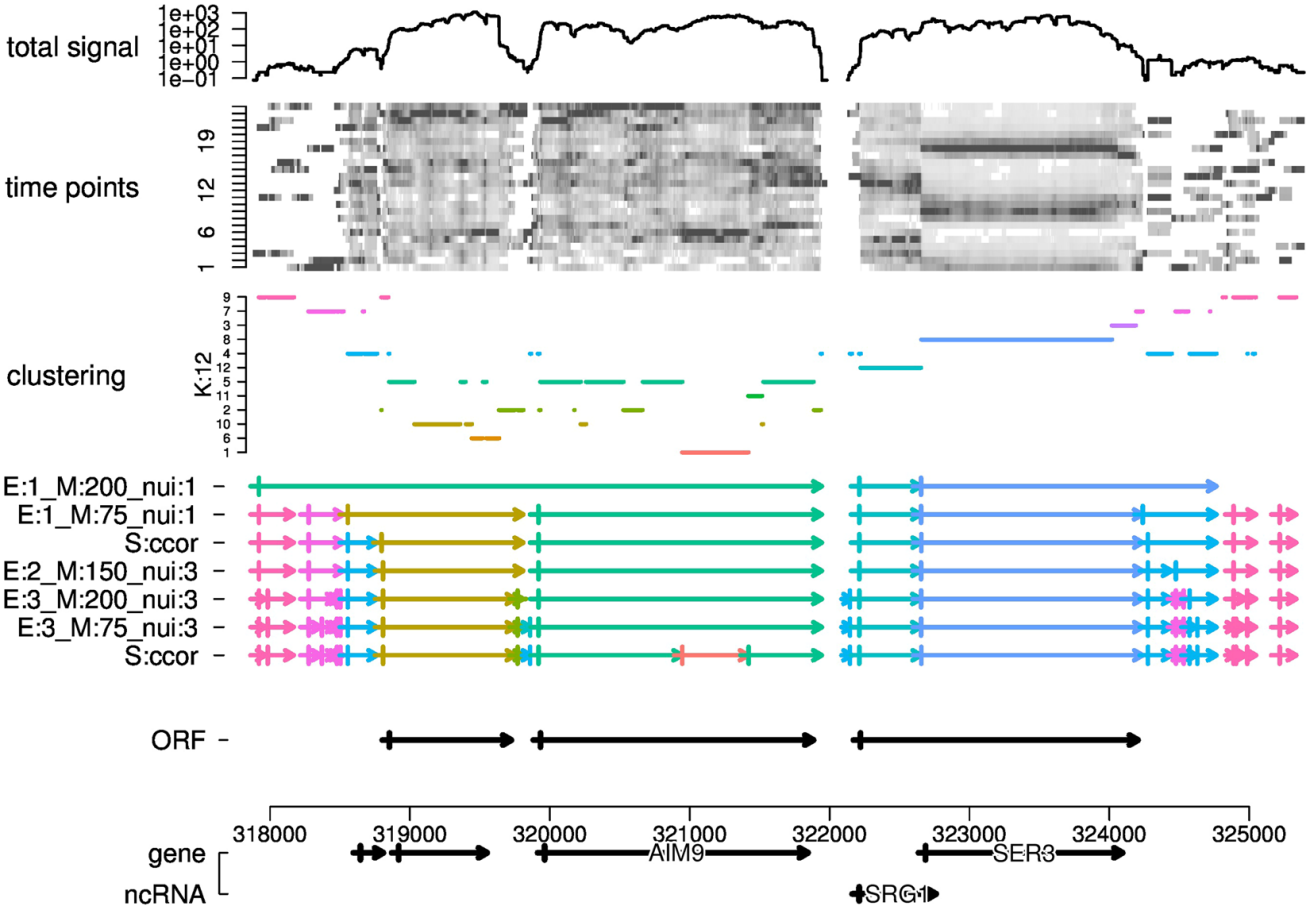
Example Region: SRG1 vs. SER3 (chrV:317829..325452). All segmentations were calculated from the shown clustering (*K* = 12, clusters sorted by similarity) of selected DFT components of the raw read-count time-series, with the indicated parameters E, M and nui, and with scoring function icor (corresponding from top to bottom to 1–5 in Fig. 2d), except where indicated (‘S:ccor’), which were calculated with the same parameters as the segments directly above but using scoring function ccor (‘x’ in Fig. 2d). ‘ORF’ are annotated transcripts from the ORF-T test set^26^, ‘gene’ and ‘ncRNA’ are annotations from the yeast genome release R64.1.1 of the actual ORF, from left to right YER078W-A, YER079W, AIM9 and SER3, and the ncRNA SRG1. This figure was produced by the demo segment_data of the segmenTier R package.

For each target segment we counted all overlapping query segments (${n}_{hits}$), then selected the query segment with the highest Jaccard index $J=|Q\cap T|/|Q\cup T|$ and calculated the query/target length ratio $R=|Q|/|T|$. To characterize a segmentation globally, we used the fraction ${R}_{short}$ of segments with $R\le 0.8$ and ${R}_{long}$ with $R\le 1.2$ (Fig. 2b), the total Jaccard index ${J}_{tot}$ of all best hits queries for all targets, and ${\tilde{n}}_{hits}$, the average number of query hits per target (Fig. 2d). A segmentation that recovers the test set well, will have high ${J}_{tot}$ and ${R}_{long}$, and low ${\tilde{n}}_{hits}$ and ${R}_{short}$.

We tested 540 parameter combinations against the ORF-T test set, varying time-series pre-processing, clustering and segmentation parameters. The segmentations were clustered by the four optimization parameters (coloring in Fig. 2) using’partitioning around medoids’ (PAM). Enrichment of these clusters with run parameters was established by Fisher’s exact tests (Fig. 2e) and provides an overview of the effects of parameters. A detailed inspection of parameter combinations in the optimal clusters (Figs 2f and S3) finally guided the choice of three optimal and two bad-scoring parameter sets for discussion, indicated as 1–5 in Fig. 2d.

## Results and Discussion

We have shown here that segmentation along a linear coordinate axis can be modeled as a combinatorial optimization problem independently of the data type or types. Indeed, the only mathematical structure required in the data domain is a symmetric similarity measure that favors identity, i.e., satisfies conditions (s1) and (s2). An efficient implementation is made available with the R package segmenTier.

As a showcase application we consider the segmentation of a time-series of 24 yeast transcriptomes, taken over 2.5 cycles of the respiratory oscillation (Supp. Fig. S2). The rich transcriptome dynamics of this experimental system are well explored by hybridization based techniques^21–23^, which, however, only measure known protein-coding transcripts. RNA-seq allows to establish transcription at single nucleotide-resolution for the whole genome, and thus evaluate also the extent and dynamics of non-coding or antisense transcription, which is widely thought to have regulatory functions or encode for functional RNA molecules. While RNA-seq experiments had also been performed for this oscillatory system, the sequencing was not strand-specific and analyzed only on a per-gene level^46,47^.

Using a pre-segmentation at large non-expressed regions (Fig. 1) segmenTier computes segmentation of the complete yeast genome data set in about 15 hours on a desktop computer (Lenovo ThinkCenter, Intel i7-4790 CPU (3.60 GHz), Fedora F22). This performance made is possible to explore the parameter space systematically.

### ORF Transcript Recovery

To our knowledge no tools with comparable functionality, *i.e*., segmentation of RNA-seq time-series data based on consistent temporal profiles, exist. Thus, we could not compare the performance of the algorithm directly with other tools. Instead, we reasoned that previously annotated protein-coding transcripts should also be expressed as temporally coherent segments in our data set. We systematically scanned 540 parameter combinations and tested the resulting segments for recovery of a set of 5171 published transcripts^26^ annotated as Open Reading Frame transcripts (ORF-T) (Fig. 2). Over-fragmentation is observed when segments are shorter than target ORF-T and a given ORF-T comprises several segments, and under-fragmentation when adjacent segments are not split (Fig. 2b and d).

The test revealed the exponent $\varepsilon$, the nuisance cluster similarity $\nu$ and the length-penalty score $M$ as major determinants of ORF-T recovery performance. Notably, a good recovery of ORF-T can be achieved with different opposing combinations, combining either higher $M\ge 150$ with $\varepsilon  > 1$ and $\nu  > 1$, or low $M < 150$ and $\varepsilon =\nu =1$ (Fig. 2e and f). We also observed small but systematic effects of the of choice of data pre-processing, the number of clusters $K$, and scoring function $S$ (Supp. Fig. S3a) which contributed to our final choice of parameter sets used for further discussion and indicated in Fig. 2d. Parameter sets 1, 3 & 5 recovered ORF-T with total Jaccard indices ${J}_{tot}$ of $\mathrm{53 \% }$, $\mathrm{75 \% }$ and $\mathrm{66 \% }$ (Fig. 2d), $\mathrm{35 \% }$, $\mathrm{64 \% }$ and $\mathrm{49 \% }$ of ORF-T with a Jaccard index $J > 0.8$ (Fig. 2c, vertical line at 0.8), and $80$ % of ORF-T with $J > 0.32$, $J > 0.62$ and $J > 0.50$ (Fig. 2c, horizontal line at 0.2).

### Example Transcripts

**Figure 4:**
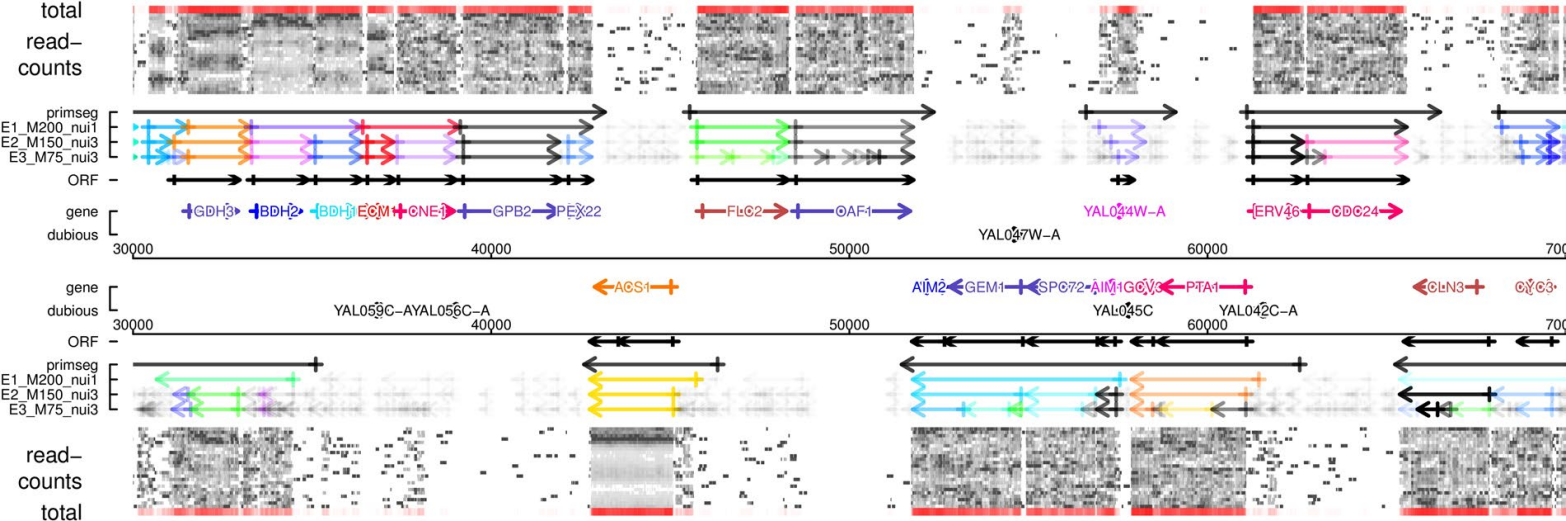
Example Domain (chrI:300000..70000). A densely transcribed domain from chromosome I is shown, transcribed segments are shown separately and in reverse order for the forward (top) and reverse (bottom) strand. ‘total’ is a color-coded version of the total signal (sum over all time points), ‘read-counts’ are the individual time-points, ‘primseg’ are the primary segments which were used to split segmentation (Fig. 1), the shown segmentations are from top to bottom those indicated as 1, 3 and 5 in Fig. 2d. Segments were colored by calculating a cycle phase from the average segment read-count along a color wheel (Supp. Fig. S5), and color alpha was derived from a p-value ($-{\mathrm{log}}_{2}(p)$) for oscillation calculated by the rain R package^49^. Segments with $p\ge 0.05$ are shown in black, where the alpha is derived from their total expression level. ‘ORF’ are the experimentally defined ORF-T transcripts from^26^ which were used in the parameter scan (Fig. 2), ‘gene’ and ‘dubious’ are annotated confirmed and dubious open reading frames in the yeast genome release R64.1.1. The ‘gene’ ORF are colored by a previous consensus definition of rhythmically co-transcribed gene cohorts in this experimental system^23^. The plot was generated using the genomeBrowser R script collection at https://gitlab.com/raim/genomeBrowser.

The detailed effects of parameters can be illustrated on specific examples: a small region from chromosome V comprises of four ORF and one non-coding RNA (Figs 3 and S5), and a broader genomic region (Fig. 4). The non-coding RNA SRG1 regulates the transcription of the downstream overlapping SER3 ORF transcript via a transcription-interference mechanism^48^.

$M$ allows to enforce minimal length requirements for the expected signal, here RNA transcripts. However, unlike most eukaryotic genomes the yeast genome is highly compact and densely packed with transcripts (Fig. 4). We often observe a gradient of signal towards the end of transcriptional units, indicating the distribution of actual transcription start and end sites or potential read-through events. Thus, the signal between distinct transcriptional units often remains above 0 and often will reflect contributions from both transcripts. For example the three transcripts of genes YER078W-A, YER079W, AIM9, upstream of the SRG1, remain un-segmented with parameters $\varepsilon =\nu =1$ at $M=200$ (Figs 3 and S5a). Amplifying or scaling of the cluster-position and nuisance similarity measures by $\varepsilon$ and $\nu$ allows to locally overcome length requirements set by $M$ and cut-off small and mostly noisy segments between adjacent and at the end of transcripts. Several such examples can be observed in Fig. 4.

In contrast, the overlapping transcriptional units of SRG1 ncRNA and SER3 ORF transcript both have a strong and distinct signal. Their detection is stable over the parameter range tested (Fig. 3). It is notable that this pair was not identified as separate transcripts in the ORF-T data set. The genome contains several known and expectably many more unknown examples of such overlapping transcripts interacting by transcriptional interference mechanisms, and potentially un-detected in our ORF-T set. Such artifacts may in part explain why the Jaccard measures $J$ and ${J}_{tot}$ remain well below their maximum (Fig. 2c and d).

Setting a lower $M$, combined with similarity scaling often leads to internal breaks that appear artefactual but do usually represent subtly distinct temporal profiles, e.g., the central portion of gene AIM9 is split at $\varepsilon =\nu =3$ and $M=75$ with scoring function ccor (Fig. 3, bottom segmentation). It is for the present analysis and without further experimental tests impossible to judge all individual break points, several experimental and data processing sources of noise can bias local signals. But it is well understood that “cryptic” transcription can occur inside of and overlapping with more traditionally defined transcriptional units^26,27^. Optimal parameter settings will highly depend on the data structure and the purpose of analysis. Parameters could be geared to higher break sensitivity to analyze more subtle signals, e.g. from splice sites, or merely pinpoint methodological artifacts.

User-defined choices of parameters cannot be entirely avoided in segmentation problems. This is in particular true in applications where the structure of the data domain is either unknown or deliberately modeled with little internal detail. In most cases the similarity measure will be defined heuristically based on the user’s intuition about the data or based on a prior empirical exploration. For our show-case example, oscillatory transcriptome dynamics, we had previously observed that the phase and amplitude information describes the data better than the more obvious representation of the time-series as vectors in a euclidean space^23,28^. We note that in the context of the segmentation problem, it proved useful to additionally account for the total coverage to achieve better segmentation between close adjacent fragments. This seems to reflect the tendency of adjacent genes to show correlated expression patterns^50^.

The *segmenTools* package therefore has been designed to make it easy to scan and evaluate the effects of parameters. It also implements optimization strategies for the parameters to be used in applications where an annotated set of test data is available.

The clustering techniques available for similarity spaces typically contain a random component, such as the choice of the initial medoids in the $k$-medoids approach. The efficiency of segmenTier makes is possible to compute multiple segmentations so that the robustness of the segmentation can be determined. This also suggests to investigate, in future research, systematic methods to define and compute consensus segmentations. A simple majority voting over several runs with identical parameters but exploiting the random effect of $k$-means clustering, may already yield good results (Supp. Fig. S4b).

### Concluding Remarks

The general 1D segmentation method outlined here poses several questions for future research. Although we have shown that the results are quite robust over a range of parameter values for data as complex as the yeast transcriptome, some parameters choice have to be left to user as matter of design: most importantly, the user has to supply an estimate of the typical segment size or an estimate of the noise level. Without any prior knowledge, these cannot be estimated from the data as a matter of principle. To see this, suppose that there is no noise at in the data. Then every jump in the data is real and segments are interval of exactly constant signal. In the other extreme, the input might be entirely noise and the correct answer is a single segment.

The second key parameter is the number of clusters $K$ that are to be used. As we have seen, the segmentation problem can be phrased without a clustering step. For most applications, however, this is beyond the limits of computational resources. We have seen above that $K$ only has a moderate influence on the results. If $K$ is chosen much too large, this mostly infringes on the computational efficiency. Too few clusters, on the other hand, may lead to incomplete segmentation. Classical information-theoretic methods^51^ or the silhouettes method^52^ are certainly applicable to estimate good values of $K$, or even assess the quality of the re-clustering of the data set by the segmentation. It remains a question for future research, however, whether values of $K$ optimal in terms of clustering also yield the best segmentation results.

An important issue is the robustness of the segmentation. As the most simple test we have used independent instances of $k$-means clustering to assess this issue. One might also consider bootstrap-like re-sampling. The construction of multiple segmentations for the same data set, either due to randomized components in the clustering algorithm (here the random choice of the $K$ initial centroids), or due to variations in the parameters, naturally leads to the question how segmentations should be compared and how to define a consensus set of segmentations. In particular the latter appears to be an interesting problem for future research that is not only of relevance for the future releases of segmenTier but also for problems such as the reconciliation of multiple chromatin segmentations.

### Availability

The software is available as an R package from https://github.com/raim/segmenTier.

## Electronic supplementary material

Supplementary Information
