# Supplementary material for: Similarity-Based Segmentation of Multi-Dimensional Signals
Source: Sci Rep. 2017 Sep 27;7:12355. doi: 10.1038/s41598-017-12401-8 (PMC5617875; doi:10.1038/s41598-017-12401-8)
Supplement: Supplementary file 1 — Supplementary Information [file 41598_2017_12401_MOESM1_ESM.pdf]

Similarity-Based Segmentation of Multi-Dimensional Signals
Appendix A & Supporting Figures S1–S5

Rainer Machné, Douglas B. Murray and Peter F. Stadler

June 4, 2017

Appendix A

Let A be a symmetric $n \times n$ matrix. In order to compute $B_{k,l} := \sum_{i=k}^{l} \sum_{i=k}^{l} A_{ij}$ and $M_{k,l} := \max_{k \leq p \leq l} \sum_{i=k}^{l} A_{ij}$ efficiently, we may construct the auxiliary matrices U and V in quadratic time:

$$U_{ip} := \sum_{j=1}^{p} A_{ij} = U_{i,p-1} + A_{ip}$$

$$V_{rp} := \sum_{i=1}^{r} \sum_{j=1}^{p} A_{ij} = \sum_{i=1}^{r} U_{i,p} = V_{r-1,p} + U_{r,p}$$

with initial conditions $U_{i1} = A_{i1}$ and $V_{1p} = U_{1p}$. Then $B_{kl} = V_{ll} + V_{k-1,k-1} - 2V_{k-1,l}$ and $M_{kl} = \max_{k \leq p \leq l}(U_{pl} - U_{p,k-1})$. This B and M can be computed in quadratic and cubic time, respectively.

Supporting Figures

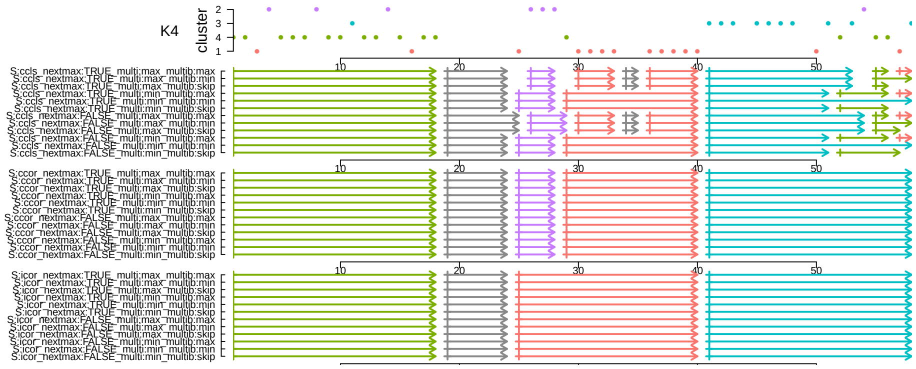

Figure S1: **Simulated Data.** Only scoring function `ccls` is sensitive to internal details of scoring function calculation and back-tracing.

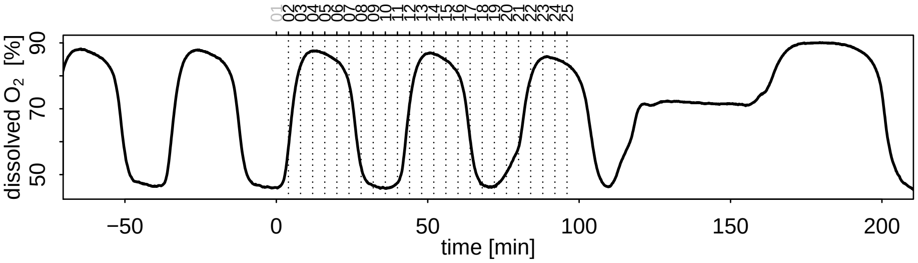

Figure S2: **Sampling of the RNA-seq Time-Series: Dissolved O_2.** The experimental system, respiratory oscillations during continuous growth of budding yeast strain IFO 0322, has been described extensively (Murray *et al.*, 2007 [39], Machné & Murray, 2012 [23]) growth and dynamics of dissolved Oxygen in the reactor were equivalent to previous experimental conditions (Murray *et al.*, 2007 [39]), and oscillation appeared stable. Samples 01–25 were taken at the times indicated, sample 01 was discarded and only samples 02–25 were processed. Notably, the culture underwent a transient shortly after sampling, perhaps induced by sampling itself, then re-established oscillatory growth. Reference numbers refer to the main article.

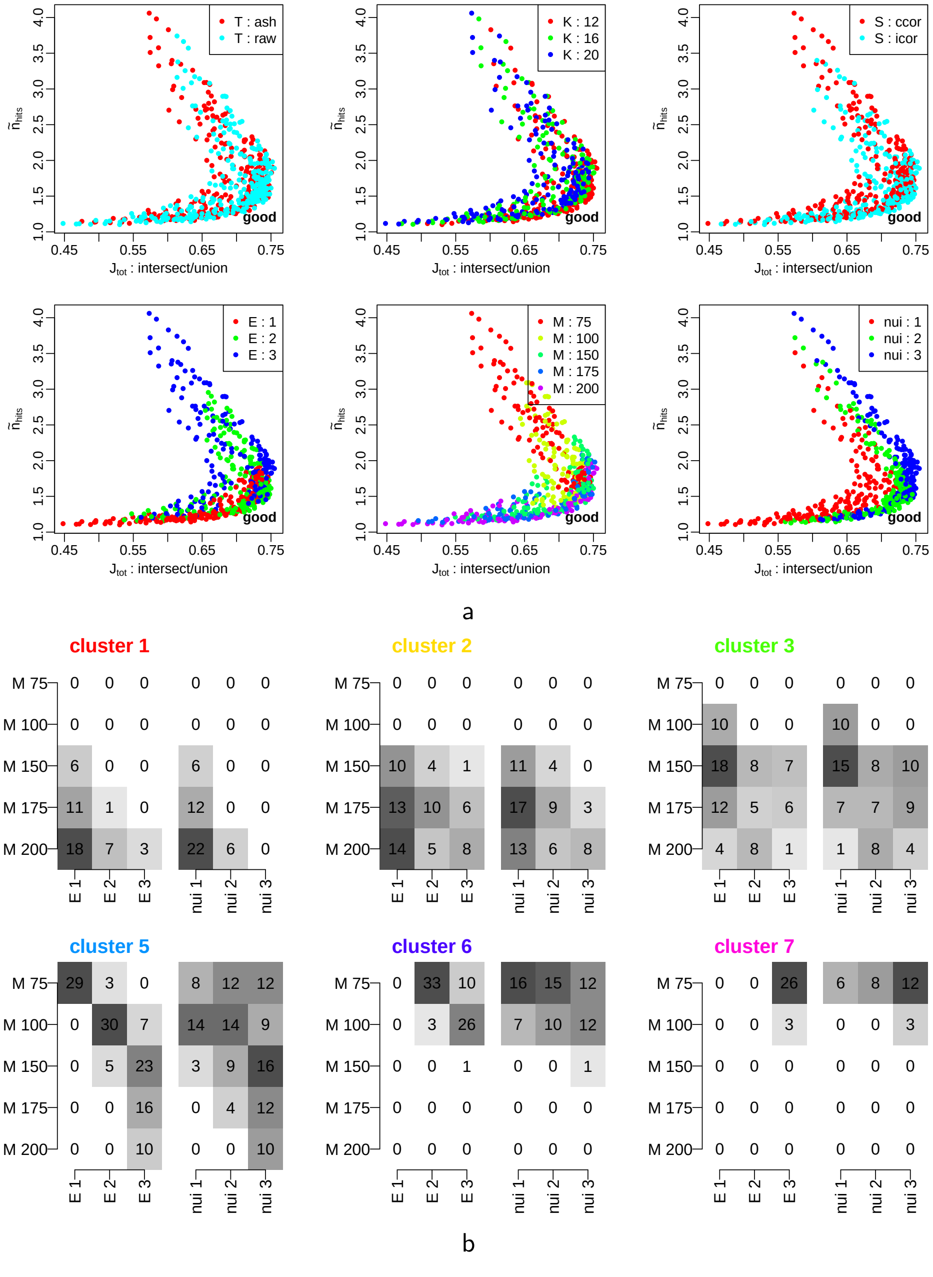

Figure S3: **Parameter Scan: Parameter Effects.** (a) As Figure 2d of the main article, but colored by time-series processing, clustering and segmentation parameters. (b) As Figure 2f but for all other PAM clusters shown in Figures 2b–2e.

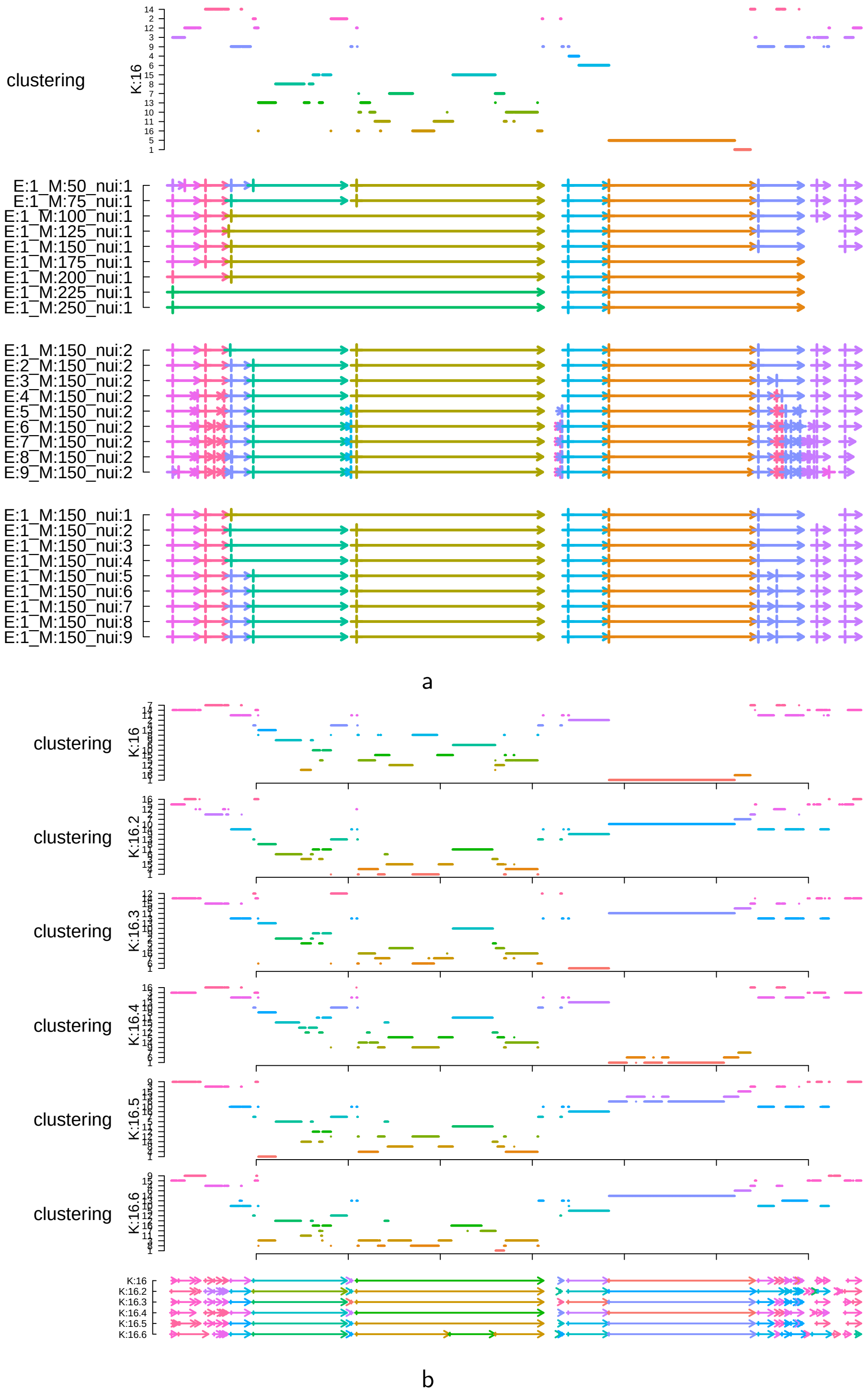

Figure S4: **Example Region: SRG1 vs. SER3.** Systematic scans of dominant parameters (a) and random effect from cluster initialization (b).

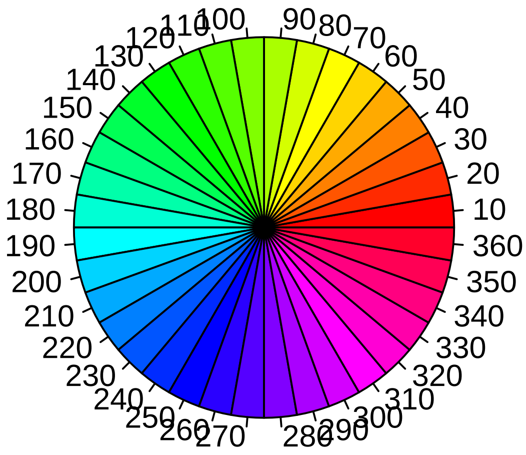

Figure S5: **Phase coloring scheme.** Phase coloring scheme used in Fig. 4 of the main article.
